# Analysis of the neuroprotective effect of GLP‐1 receptor agonist peptide on cerebral ischemia‐reperfusion injury by Quantitative Proteomics Mass Spectrometry

**DOI:** 10.1002/brb3.2190

**Published:** 2021-05-21

**Authors:** Ying Li, Min Gong

**Affiliations:** ^1^ Tianjin Neurological Institute Tianjin Medical University General Hospital China; ^2^ Department of Pharmacy Tianjin Medical University Tianjin China

**Keywords:** cerebral ischemia reperfusion injury, proteomics

## Abstract

**Objective:**

The pathological characteristics of cerebral ischemia‐reperfusion injury (CIRI) are complex, and the mechanism involved remains unknown. The treatment for CIRI has become an increasingly important challenge in the clinic, prompting us to explore the mechanism of CIRI. It was reported that GLP‐1 receptor agonist, Liraglutide, exhibited alleviating effects on CIRI. The previous findings suggested that the administration of Liraglutide in rodents results in the attenuation of the infarct volume following ischemia‐reperfusion injury by mediating the reactive oxygen species, apoptotic and necroptotic pathways.

**Methods:**

Here, a proteomic study was performed aiming to clarify the physiological protection role of GLP‐1 receptor agonist during the development of CIRI in MCAO mice. This proteomic investigations is contributed to reveal the mechanism associated with the treatment of GLP‐1 receptor agonist in MCAO mice.

**Results:**

The results indicated that the occurrence of ischemia‐reperfusion led to complex pathological processes, including inflammation, necroptosis and apoptosis. The treatment of Liraglutide significantly reduced the infract volume resulted from ischemia reperfusion injury. The proteomic data revealed that the administration of Liraglutide in MCAO mice induced the various effects on proteins expression level and phosphorylation.

**Conclusions:**

The findings in this study was beneficial for identifying the novel therapeutic target for the treatment of ischemia reperfusion.

## Graphical abstract

Liraglutide, a GLP‐1 receptor agonist, effectively reduced the infarct volume in the brain in MCAO mice. The physiological effects of liraglutide in ischemia‐reperfusion injury were dependent on the suppression of oxidative stress, on the inhibition of neuronal apoptosis and a reduction in the inflammatory response.

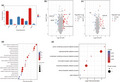

## 1 INTRODUCTION

Cerebrovascular disease is a common risk factor for disability in the elderly, especially cerebrovascular ischemic disease (Caplan et al., 2009). Cerebral ischemia‐reperfusion injury (CIRI) leads to severe brain dysfunction, even when the blood supply is restored after a certain period of cerebral ischemia (Randolph, 2016). Despite the severity of cerebral ischemia, the only currently available FDA‐approved pharmacological intervention for ischemic stroke is recombinant tissue plasminogen activator (rtPA), the use of which is complicated by the relatively short window of time between infarct and treatment (3–4 hr) and an increased risk of subarachnoid hemorrhage (Micieli et al., 2009). CIRI has become an increasingly important challenge related to the treatment of cerebrovascular diseases by endovascular therapies, including thrombectomy and thrombus destruction (Abbott et al., 2010). Recently, many other agents developed to slow or prevent the occurrence of CIRI have exhibited promising efficacy in the initial stage of research but have thus far failed in further clinical trials. Unfortunately, to this point, no pharmacological interventions have been shown to be efficacious in human clinical trials. A new approach for investigating the mechanisms of cerebral ischemia‐reperfusion injury is, therefore, needed. Recent evidence has shown that the complex mechanisms of cerebral ischemia‐reperfusion injury included oxidative stress (Wu et al., 2020), leukocyte infiltration (Barone et al., 1995; Feng et al., 2017), platelet adhesion and aggregation (Li et al., 2015; Sugidachi et al., 2016), complement activation, mitochondrial dysfunction (Imai et al., 2020; Li et al., 2014), and blood‐brain barrier (BBB) damage, which ultimately lead to nerve cell death and brain tissue damage (Abdullahi et al., 2018).

Recently, it was demonstrated that Liraglutide, a glucagon‐like peptide‐1 receptor (GLP‐1R) agonist is capable to retard the progression of CIRI (Deng et al., 2018; Nauck et al., 2017; Sato et al., 2013). Liraglutide was considered to possess a neuroprotective effect by inhibiting neuronal apoptosis (Briyal et al., 2014), suppressing oxidative stress (Briyal et al., 2014) and improving mitochondrial function (Zhu et al., 2016). GLP‐1R agonists have recently been reported to protect against ischemic stroke by reducing the degradation of tight junction proteins (TJPs) between endothelial cells to maintain BBB stability in both diabetic and non‐diabetic middle cerebral artery occlusion (MCAO) models and a model of traumatic brain injury (Fukuda et al (2016); Hakon et al (2015)). However, the mechanism by which GLP‐1R agonists alleviated ischemia‐reperfusion injury remained unclear (Dong et al., 2017).

Proteomic analysis was performed in this assay to investigate the alterations on protein expression patterns after ischemia‐reperfusion injury upon the treatment of Liraglutide. The findings of this study are helpful for elucidating the mechanism underlying the effect of GLP‐1R agonists during the progression of ischemia‐reperfusion injury and may contribute to the development of GLP‐1R agonists as systematic therapeutic agents for CIRI in the clinic.

## 2 MATERIALS AND METHODS

### 2.1 Animals and treatments

Twenty‐four C57BL/6J mice (male, 7–8 weeks old) were purchased from Tonglihua Company (Beijing, China). The animals were divided into the following groups: saline‐treated mice subjected to MCAO (the CK group), mice treated with Liraglutide (6 μg/kg/4 hr) after MCAO (the D.M group), and sham group (the NS group with MCAO). The animal experiments were carried out in accordance with the guidelines of the China Animal Care and Use Committee of Tianjin Medical University.

### 2.2 Construction of animal model of MCAO

The all animals were subjected to MCAO (middle cerebral artery occlusion) to construct an animal model of CIRI. The mice were intraperitoneally injected with 3% pentobarbital sodium (80 mg/kg). The common carotid artery, internal carotid artery, and external carotid artery were separated after the skin on the head was prepared and disinfected. Briefly, a monofilament (0.18 mm) was introduced into the common carotid artery under anesthesia, advanced to the origin of the MCA, and left in place for 2 hr until reperfusion. After 2 hr of ischemia, the filament was removed, and the skin was sutured. The mice were placed on a heating pad and returned to their home cages after they awoke. The brains of the anesthetized mice were collected 48 hr after reperfusion for further experiments. The sham operation was performed following the same procedure, but the surgery was stopped after the dura mater was opened.

### 2.3 TTC staining

The mice were deeply anesthetized with sodium pentobarbital (80 mg/kg). The brains were removed quickly (within 10 min) and transferred to a −20°C freezer for 30 min. Coronal brain sections (2 mm thick) were stained with 2% red tetrazolium solution (Sigma‐Aldrich, USA) for 30 min at 37°C in the dark. The container was slightly shaken every 5 min to ensure complete staining. The brain slices were washed with PBS for 3–5 min and then fixed with 10% neutral formaldehyde for 6 hr, after which images were immediately captured.

### 2.4 Protein extraction

Tissue samples were weighed and ground after the animals were euthanized. Subsequently, the samples were lysed in lysis buffer (8 M urea and 1% protease inhibitor cocktail) and ultrasonicated. The cell debris was removed by centrifugation at 12,000 × g for 10 min, and then, the protein concentration was determined using a BCA kit (Pierce, Thermo Scientific). Before trypsin digestion, the protein samples were treated with dithiothreitol (5 mM) for 30 min at 56°C to reduce the urea concentration, and then, the solution was alkylated with iodoacetamide (11 mM) for 15 min at room temperature in the dark. Finally, trypsin digestion was performed according to the manufacturer's instructions.

### 2.5 TMT labeling, liquid chromatography‐mass spectrometry (LC‐MS), and analysis

TMT (tandem mass tag) labeling was first performed by PTM Biolab Service (Hangzhou, China) using a TMT kit. Briefly, the peptide mixtures were then incubated with one unit of TMT reagent for 2 hr at room temperature and dried by vacuum centrifugation after desalting.

The tryptic peptides were fractionated by reverse‐phase HPLC using a Thermo Betasil C18 column (5 μm particles, 10 mm ID, 250 mm length). The collected peptide fractions were then dissolved in 0.1% formic acid (solvent A) and directly loaded onto a homemade reversed‐phase analytical column (15 cm length, 75 μm i.d.). The gradient comprised an increase from 6% to 23% solvent B (0.1% formic acid in 98% acetonitrile) over 26 min, an increase from 23% to 35% over 8 min, an increase to 80% over 3 min and then a hold at 80% for the last 3 min, all at a constant flow rate of 400 nl/min on an EASY‐nLC 1,000 UPLC system.

### 2.6 Western blot

Proteins were separated by 10% sodium dodecyl sulfate‐polyacrylamide gel electrophoresis (SDS‐PAGE), and then, immunoblotting with a mouse anti‐hippocalcin antibody (Abcam), a mouse anti‐neuromodulin antibody (Abcam), and a horseradish peroxidase‐conjugated goat anti‐rabbit IgG secondary antibody (Abcam) was performed.

### 2.7 Data analysis

The resulting MS/MS data were processed using the Maxquant search engine (v.1.5.2.8). Tandem mass spectra were searched against the human UniProt database concatenated with the reverse decoy database. Trypsin/P was specified as a cleavage enzyme allowing up to 4 missing cleavages. The mass tolerance for precursor ions was set as 20 ppm in the first search and 5 ppm in the main search, and the mass tolerance for fragment ions was set as 0.02 Da. Carbamidomethyl on Cys was specified as a fixed modification, and acetylation modification and oxidation on Met were specified as variable modifications. The FDR was adjusted to <1%, and the minimum score for modified peptides was set to >40. Subsequently, bioinformatics analysis was performed using an online algorithmic database (UniProt‐GOA, InterPro, KEGG Orthology, Wolfpsort, and InterPro).

All measurements were taken from samples chosen based on study feasibility and analyzed using SPSS 22.0 software. Differences between the control and MCAO groups for normally distributed variables were tested using Student's *t* test. The data are expressed as the mean ±standard deviation. Differences were considered significant at a *p* value<.05.

## 3 RESULTS

**FIGURE 1:**
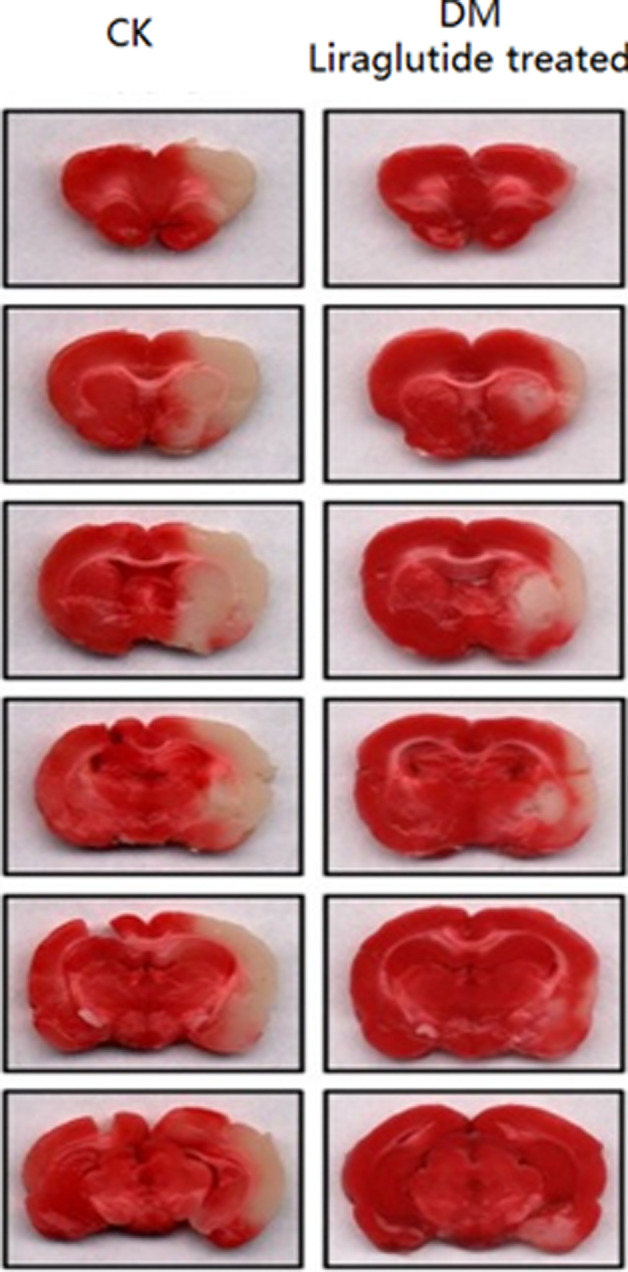
TTC staining. The TTC staining results indicated that Liraglutide significantly reduced the infarct volume, *n* = 6

The results showed that administration of Liraglutide significantly alleviated the infarct indicating that Liraglutide played essential physiological roles to ameliorate the injury resulted from MCAO (Figure 1).

**FIGURE 2:**
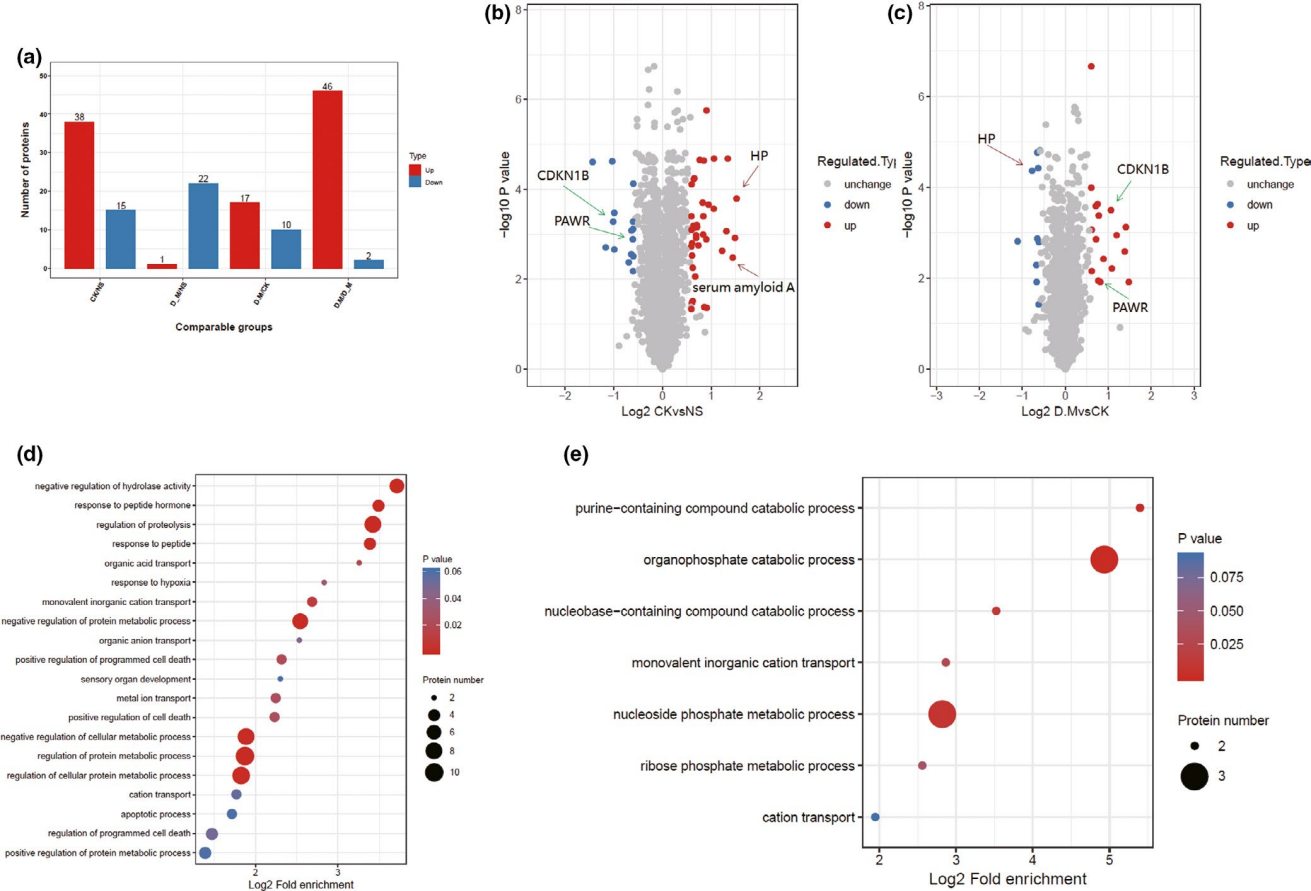
Functional enrichment analysis of differentially expressed proteins in MCAO mice treated with/without Liraglutide (control group, *n* = 4; MCAO, *n* = 4). Volcano plot showing the effect of Liraglutide on differentially expressed proteins in MCAO mice (FDR <0.01). Significantly differentially expressed proteins are color‐coded: red indicates up‐regulated proteins, and blue represents downregulated proteins

The further proteomic data revealed that a total of 27 DEPs (differentially expressed proteins) were found in the mice treated with Liraglutide, including 17 up‐regulated proteins and 10 downregulated proteins, in comparison with that of saline‐treated mice (Figure 2). It was found that Liraglutide inhibited the neuronal apoptosis following MCAO/R injury by downregulating the expression of Rho‐associated protein kinase 1, Bcl‐2, Bax, and other proteins. It was also found that the expression levels of several proteins were significantly altered between the DM group (Liraglutide‐treated mice subjected to MCAO) and CK group (MCAO mice) and NS group (sham group). For example, the protein expression of PRKC apoptosis WT1 regulator (PAWR, also named Par‐4) was markedly downregulated (CK group/NS group expression ratio of 0.54) upon the occurrence of ischemia‐reperfusion, suggesting that the expression of PAWR was reduced in MCAO mice compared with sham mice (Table 1). However, PAWR protein expression was up‐regulated in the DM group compared with the CK group (DM group/CK group expression ratio of 1.76), suggesting the expression was enhanced in Liraglutide‐treated animals compared with saline‐treated MCAO mice.

**TABLE 1:** Several distinct DEPs (Differentially Expressed Proteins) in the progression of ischemia‐reperfusion with/without Liraglutide treatment

Protein ID	MW	CK/NS value	DM/CK value
PAWR	35.908	0.504 (down)	1.76 (up)
HP	38.752	2.878 (up)	0.585 (down)
SAA	13.77	2.722 (up)	‐‐‐‐
CDKN1B	22.193	0.497 (down)	2.088 (up)

Note

CK group: saline‐treated mice with MCAO; NS group: sham group; DM group: Liraglutide‐treated mice with MCAO.

The proapoptotic protein PAWR is capable of inducing apoptosis of cancer cells, sensitizing cancer cells to diverse apoptotic stimuli and causing the regression of tumors in animal models (Chakraborty et al., 2001). PAWR induces apoptosis by activating the Fas pathway and downregulates the expression of the antiapoptotic protein Bcl‐2 via its interaction with WT1 (Chakraborty et al., 2001). Interestingly, previous studies have indicated that PAWR is directly involved in regulating the BACE1‐mediated cleavage of amyloid precursor protein (APP) (Xie & Guo, 2005). However, there is no direct evidence indicating that PAWR is related to ischemia‐reperfusion injury until now.

Haptoglobin (Hp) is another protein that exhibited a distinct expression pattern in MCAO mice after Liraglutide treatment. The expression of haptoglobin was significantly up‐regulated in MCAO mice (CK group) compared with the sham group and then was downregulated in Liraglutide‐treated MCAO mice. The physiological function of haptoglobin is to eliminate free hemoglobin, and haptoglobin is also an iron‐containing protein complex that transports oxygen throughout the body (Spagnuolo et al., 2014). ELISA and Western blotting revealed that haptoglobin levels in the rat brain increase markedly with age (Spagnuolo et al., 2014). Recently, evidence has indicated that haptoglobin is associated with vascular diseases, including ischemia‐reperfusion injury (Holme et al., 2009; Staals et al., 2008), but the mechanism remains unknown. It has been suggested that the expression level of haptoglobin can be employed as a biomarker for atherothrombotic ischemic stroke diagnosis since it is high upon the occurrence of ischemia‐reperfusion injury (Brea et al., 2009). Haptoglobin is recognized to play an important role in suppressing inflammatory responses since increased regulation of haptoglobin is stimulated by inflammatory responses such as infection and autoimmune reactions (Ratanasopa et al., 2013). It has been found that the up‐regulation of haptoglobin expression is associated with blood‐brain barrier dysfunction, which causes various pathological conditions, including Alzheimer's disease (AD) (Johnson et al., 1992), Parkinson's disease (PD), and Huntington's disease (HD) (Huang et al., 2011). However, the role of haptoglobin in these diseases is not yet fully understood.

Serum amyloid A protein (SAA) is another recommended biomarker for the diagnosis of ischemic stroke (Brea et al., 2009), and the current proteomic study supports this recommendation. It was found in this study that serum amyloid A expression was significantly up‐regulated (DM group/CK group expression ratio of 2.72) upon ischemia‐reperfusion but was decreased to normal levels by Liraglutide treatment. The serum amyloid A (SAA) protein is an acute‐phase reactant protein associated with high‐density lipoprotein (HDL) particles and is up‐regulated 1000‐fold during inflammation. An elegant animal experiment revealed that the infarct volume is significantly decreased in SAA‐deficient mice compared with control mice after MCAO (Yu et al., 2019). It was demonstrated that SAA might play an important role in the pathogenesis of neurological disorders by up‐regulating the expression of the cytokine interleukin‐1β (IL‐1β), which is mediated by the Nod‐like receptor protein 3 (NLRP3) inflammasome, cathepsin B, and caspase‐1 (Latz et al., 2013). Recent studies have implicated SAAs in innate immunity and various disorders; however, the precise mechanism is still unclear. Our findings indicated that treatment with Liraglutide downregulated SAA expression to baseline levels, but the underlying mechanism requires further investigation.

**FIGURE 3:**
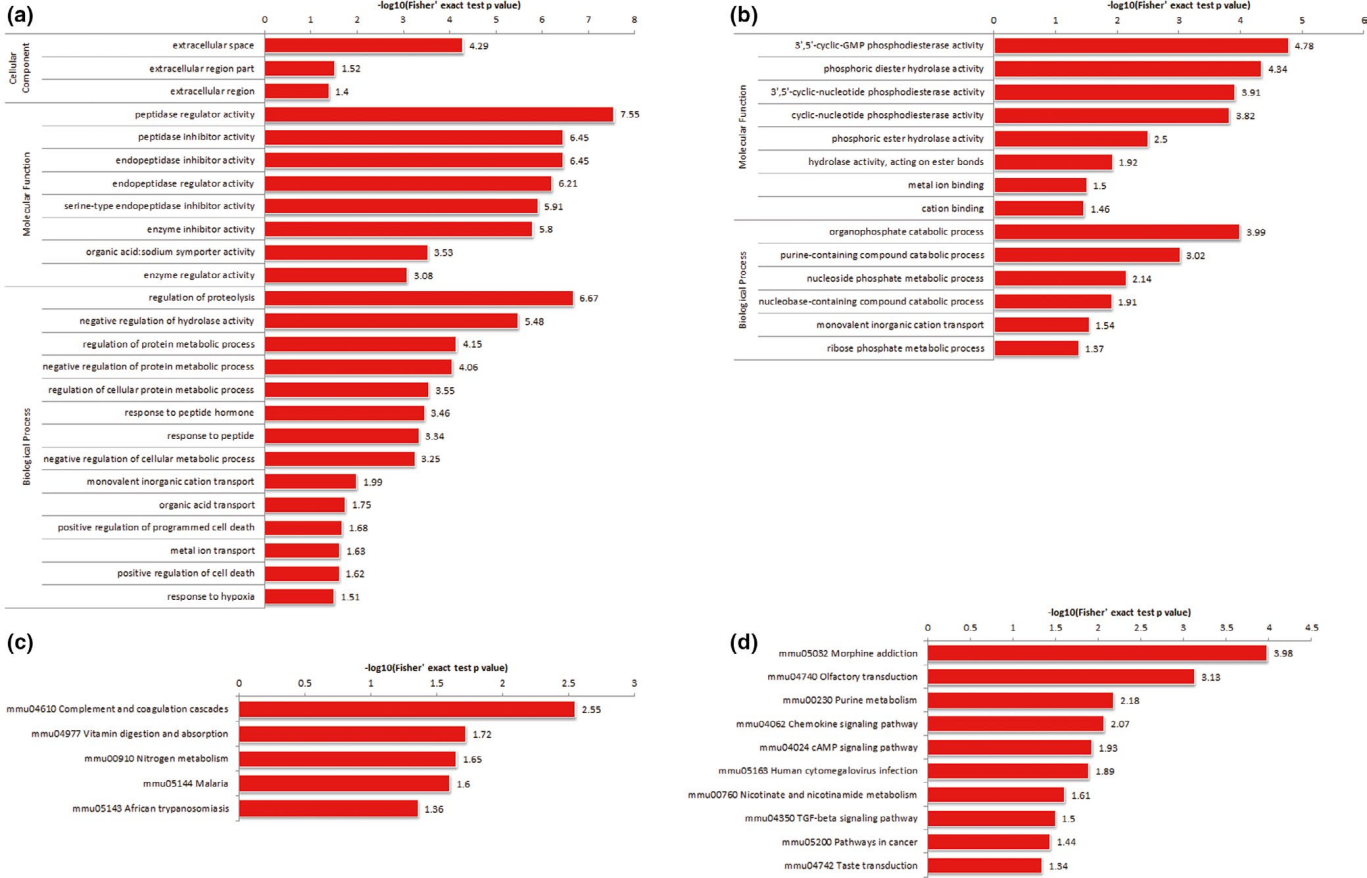
Enrichment analysis of GO biological processes and KEGG pathways in Liraglutide‐treated MCAO mice. DEP results showing the effect of Liraglutide on biological processes (BPs) and KEGG pathways. The results indicated that there were 30 GO biological processes and 26 KEGG pathways that were significantly functionally enriched

Further functional enrichment analysis identified the GO biological process (30) and KEGG pathways (26) that were significantly functionally enriched upon treatment with Liraglutide (Figure 3). Compared with MCAO mice, Liraglutide‐treated MCAO mice showed up‐regulation of various proteins related to cell component (CC), molecular function (MF), and other biological processes (BP). The expression of synapsis‐related proteins was markedly up‐regulated upon Liraglutide administration, such as Dpysl2, Syn1, Bsn, Map1b, Nf1, and Pde2a.

It was reported previously that the densities of hippocampal neurons and synapses are significantly reduced by damage to hippocampal neurons and synapses in STZ‐induced diabetic mice (Yan et al., 2019). It was found that exacerbation of oxidative stress and neuronal apoptosis might be responsible for damage to hippocampal neurons and synapses, leading to impaired learning and memory (Yan et al., 2019). However, treatment with Liraglutide obviously increased the neuronal and synaptic densities and attenuated this neuronal impairment through the PI3K/Akt signaling pathway.

**FIGURE 4:**
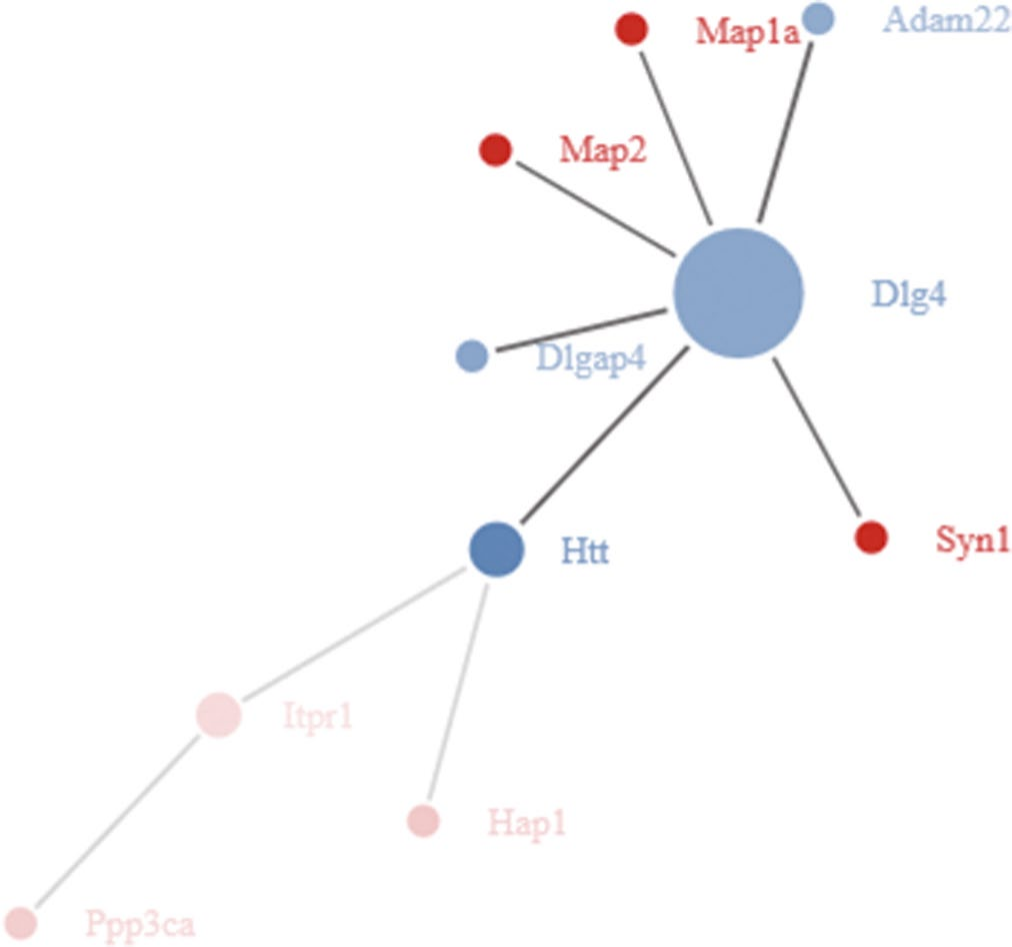
Protein‐protein interaction network in MCAO mice following the administration of Liraglutide. A total of 71 protein interaction pairs were observed in this network, which contained a total of 56 nodes. The blue nodes represent downregulated proteins, and the red nodes represent up‐regulated proteins

The interaction data revealed a total of 71 protein interaction pairs in this study. The network was constructed using Cytoscape software, and the network contained a total of 56 nodes. Node proteins with high scores included Dlg4 (degree = 10), sptan1 (degree = 8), Reps1 (degree = 5), Eif4g1 (degree = 5), Eif4b (degree = 5), Itsn1 (degree = 5), sgip1 (degree = 5), and pten (degree = 5) (Figure 4). The data indicated that the inhibitory activity of Liraglutide might be associated with the regulation of hippocampal synapses and inhibition of neuronal apoptosis.

**FIGURE 5:**
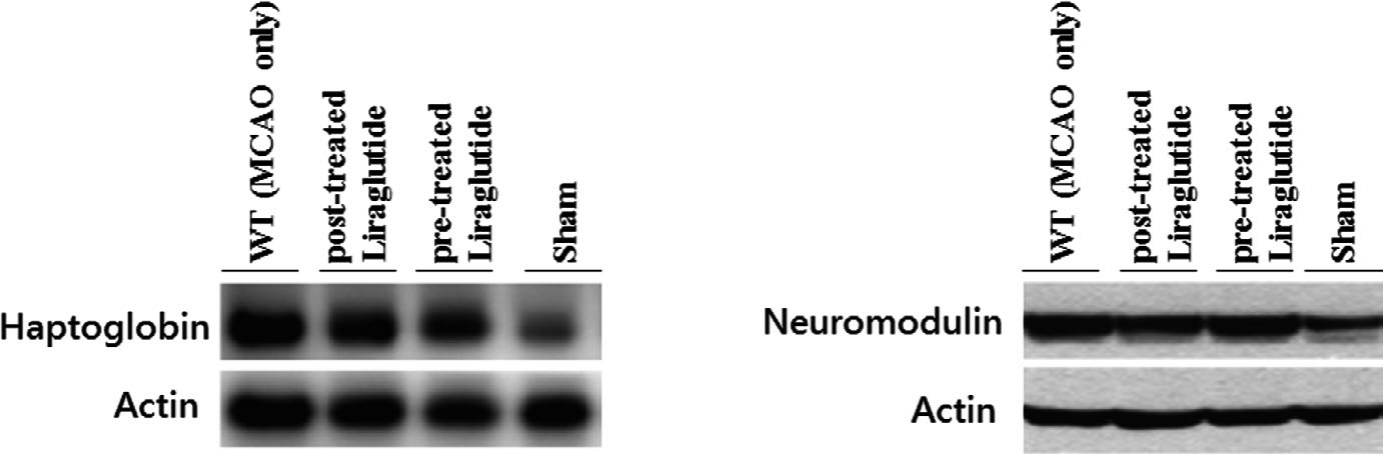
Validation of the differentially expressed proteins in the cerebral ischemia‐reperfusion animal model by Western blotting. The results indicated that the levels of haptoglobin and neuromodulin were increased by cerebral ischemia‐reperfusion. However, in Liraglutide‐pretreated mice, the levels of these proteins were downregulated, suggesting that Liraglutide had positive effects on CIRI

The Western blot images indicated that the protein expression of haptoglobin and neuromodulin was markedly up‐regulated after cerebral ischemia‐reperfusion. However, the increases in these protein expression levels were significantly alleviated in Liraglutide‐treated mice after MCAO (Figure 5).

## 4 CONCLUSION

CIRI remains an increasingly important challenge related to the treatment of cerebrovascular diseases due to its ability to induce severe brain injuries. Cell death has an important impact on the progression of brain injury after cerebral ischemia‐reperfusion injury. The inhibition of cell death in MCAO/R mice can significantly reduce the brain infarct volume. The progression of cerebral ischemia‐reperfusion injury may involve several different pathological processes from the early stage to the end stage, similar to many other diseases in the clinic.

Glucagon‐like peptide 1 (GLP‐1) is an insulinotropic hormone with glucose regulatory function (Li et al., 2011). GLP‐1 receptor is widely expressed in neurons, especially in pyramidal neurons within the neocortex (Zueco et al., 1999). Recently, it was demonstrated that a glucagon‐like peptide‐1 receptor (GLP‐1R) agonist retarded the progression of CIRI (Deng et al., 2018; Nauck et al., 2017; Sato et al., 2013). The results showed that Liraglutide, a GLP‐1R agonist, exhibited promising efficacy against CIRI in mice. However, at present, the intracellular mechanisms underlying the effects of GLP‐1 receptor agonists on cerebral ischemia‐reperfusion injury remain unclear.

Our findings indicated that the GLP‐1R agonist Liraglutide prevented oxidative damage and exerted neuroprotective activities, including by suppressing oxidative stress, promoting cell growth, inhibiting apoptosis, and reducing inflammatory responses.

Collectively, the results of this study provide a theoretical basis for further investigation of the molecular mechanisms underlying the neuroprotective effects of Liraglutide.

## CONFLICT OF INTEREST

The authors declare that they have no competing interests.

## AUTHOR CONTRIBUTIONS

Ying Li established the mouse model of MCAO construction and performed Western blot analysis. Ying Li and Min Gong wrote the manuscript.

## ETHICS STATEMENT

All animal experiments were approved by the General Hospital of Tianjin Medical University and performed following the Guide for the Care and Use of Laboratory Animals and Stroke Treatment. All authors declared NO potential conflicts of interest related to this manuscript. All authors contented this submission and publication.

### PEER REVIEW

The peer review history for this article is available at https://publons.com/publon/10.1002/brb3.2190.

## Data Availability

The datasets used and / or analyzed during the current study are available from the corresponding author on reasonable request.
